# Distribution characteristics of ABO and RhD blood groups among the voluntary blood donors in Chongqing: A retrospective study

**DOI:** 10.1097/MD.0000000000022689

**Published:** 2020-10-16

**Authors:** Hongmei Liao, Jun Li

**Affiliations:** a The Institute of Blood Transfusion; b Department of Apheresis, Component, Chongqing Blood Center, Chongqing, China.

**Keywords:** blood donor, blood donation, blood group

## Abstract

The distribution characteristics of ABO and RhD blood groups in the world were different and the data were limited. The aim was to investigate the distribution characteristics of ABO and RhD blood groups in Chongqing, and to provide scientific-effective data for a more reasonable blood collection program. We retrospectively analyzed 795 698 blood donors who had donated blood from 2014 to 2019 at the Chongqing Blood Center. The data on ABO and RhD blood groups were extracted based on blood management system. We used percentages to describe the extraction of data on blood donors by gender, age, and nationality. The data on the distribution of ABO (A, B, AB and O) and RhD (RhD-Positive and RhD-Negative) blood groups were reported also in percentages. Of those, 427 516 (53.73%) were males and 368 182 (46.27%) were females. Among all the blood donors, 321 916 (40.46%) were under the age of 25, followed by 26–35 years age group (22.65%), 36-45 years age group (18.95%), 46-55 years age group (16.98%) and 56-60 years age group (0.96%). There were 755439 (94.94%) of the blood donors who were of the Han nationality. The distribution of blood groups O, A, B and AB were 35.54%, 31.90%, 24.14% and 8.42%, respectively. The distributions of RhD-Negative group was found in 4362 (0.55%) blood donors. The distribution characteristics of the ABO and RhD blood group should be considered when improving blood collection program in Chongqing.

## 1 Introduction

The ABO and RhD blood groups, the most attention antigen of red blood cell membrane, are very important to improve the safety of blood transfusion therapy. The distribution characteristics of ABO and RhD blood groups varies in different regions and races.^[1–3]^ Earlier studies have reported the percentage of O blood group to be 34.0% in China, but 46.6% in the USA.^[4,5]^ The percentage RhD-negative blood has reported to be 1.0% in China, but 14.6% in the USA.^[4,5]^ These data on the distribution characteristics of ABO and RhD blood groups derived from blood donors in early studies. In recent years many studies have reported the distribution of ABO and RhD antigens in blood donors in local areas, but not in Chongqing. The development of economic and medical services has increased the demand for blood products. Although the continuous increases in blood collections and blood donors in China, the blood donation system still faces challenges.^[6]^ Thus, the aim was to statistically analyze the distribution characteristics of ABO and RhD blood groups in Chongqing, and to provide scientific-effective data for a more reasonable blood collection program.

## 2 Materials & Methods

We retrospectively analyzed 795,698 blood donors who had donated blood from 2014 to 2019 at the Chongqing Blood Center. The data on ABO and RhD blood groups were extracted based on the PASS SPRING SYSTEM (blood management system). This research has been approved by the Chongqing Blood Center Ethics Committee.

### 2.1 Statistical analysis

We used percentages to describe the extraction of data on blood donors by gender, age, and nationality. The data on the distribution of ABO (A, B, AB, and O) and RhD (RhD-Positive and RhD-Negative) blood groups were reported also in percentages. The data were recorded and statistical analysis in Excel 2010 (Microsoft Corporation, Redmond, WA, USA) and GraphPad Prism 6 (GraphPad Software, San Diego, CA, USA).

## 3 Results

### 3.1 Demographic characteristics of the blood donors

**Figure 1:**
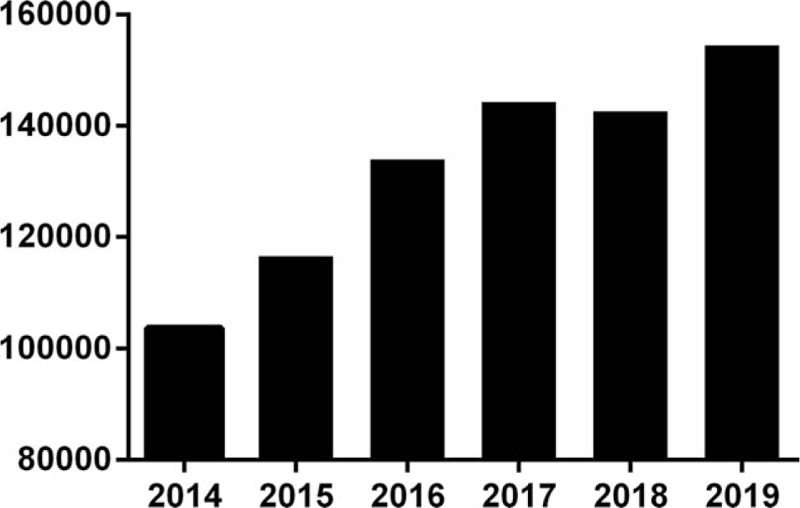
Number of blood donors in Chongqing.

We extracted data on 795,698 blood donors based on a blood management system from 2014 to 2019 (Fig. 1). Of those, 427,516 (53.73%) were males and 368,182 (46.27%) were females. (Table 1).

**Table 1:**
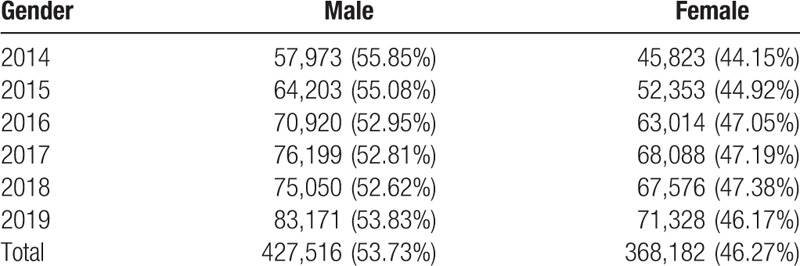
Gender distribution of blood donors in Chongqing [n(%)].

Among all the blood donors, 321,916 (40.46%) were under the age of 25, followed by 26 to 35 years age group (22.65%), 36 to 45 years age group (18.95%), 46 to 55 years age group (16.98%) and 56 to 60 years age group (0.96%) (Table 2).

**Table 2:**
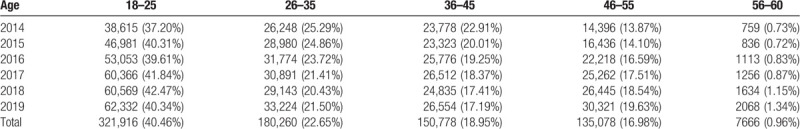
Age distribution of blood donors in Chongqing [n (%)].

There were 755,439 (94.94%) of the blood donors who were of the Han nationality. The percentages of the Tujia, Miao, Hui, Zhuang, and Yi nationality were 2.45%, 0.99%, 0.14%, 0.13%, and 0.13% respectively (Table 3).

**Table 3:**
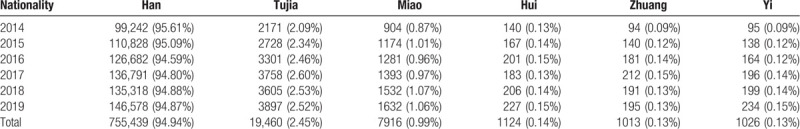
Nationality distribution of blood donors in Chongqing [n (%)].

### 3.2 Distribution of ABO/RhD blood groups

**Figure 2:**
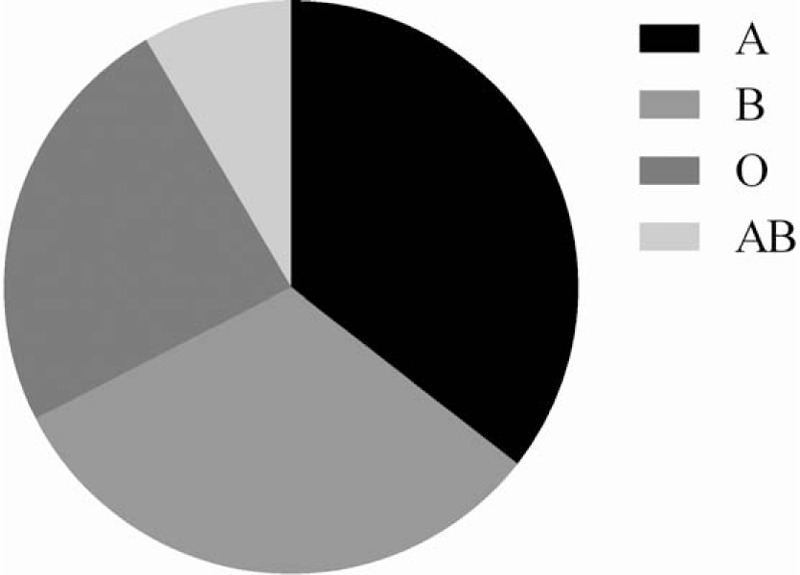
ABO blood Group distribution of blood donors in Chongqing.

The most common blood group was O group [282,812 (35.54%)], followed by A group [253,799 (31.90%)], and B group [192,120 (24.14%)]. The least common blood group was AB group [66,967 (8.42%)] (Fig. 2).

In the RhD blood groups, 0.55% (4362) of the 795,698 blood donors were RhD-Negative. The detailed data on the distribution of ABO (A, B, AB, and O) and RhD (RhD-Positive and RhD-Negative) blood groups were shown in Table 4 and Table 5.

**Table 4:**
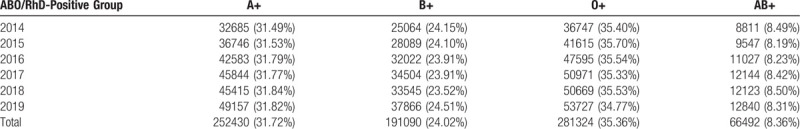
ABO/RhD-positive blood group distribution of blood donors in Chongqing [n (%)].

**Table 5:**
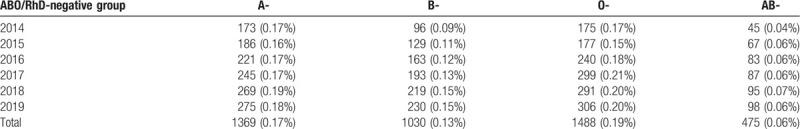
ABO/RhD-negative blood group distribution of blood donors in Chongqing[n (%)].

## 4 Discussion

The advances in medical science have increased the demand for blood products. In the past years, Chongqing has made progress on blood supply, but sometimes still faces challenges.

The discovery of ABO blood groups in 1900 by Karl Landsteiner, an Austrian pathologist, is considered as the beginning of modern blood banking and transfusion medicine.^[7]^ It should be noted that the distribution characteristics of ABO blood groups are different in different regions and races. This study on the distributions of ABO blood groups, showed a distribution of 31.90%, 24.14%, 35.54%, and 8.42% respectively for A, B, O, and AB blood groups. It involved 795,698 blood donors in Chongqing. This is consistent with previous data. In most areas of China, the distribution characteristics of ABO blood groups were O > A > B > AB.^[8]^ Similar results were seen in the studies conducted in South-West Nigeria.^[9]^ However, in other regions, such as Pakistan, B blood groups was the most common,^[10]^ while in Istanbul, the A blood groups was the most common.^[11]^ In a word, We found that the most blood donors was O blood group, followed by the A, B and AB blood groups in Chongqing.

Among the antigen of red blood cell membrane discovered so far, RhD blood groups is one of the complicated blood groups. Although transfusion therapy has saved many lives throughout the world, special blood group supply such as RhD-Negative poses risks for patients. We all know that the O RhD-Negative blood groups is a valuable resource that is often under-supplied. The previous studies have found that adequate supplying O RhD-Negative blood groups to hospital is a great challenge. Hirani showed that the demand for red blood cells declined by 21% between 2012 and 2015 in Australia, but the demand for O RhD-Negative blood groups increased significantly.^[12]^ In Chongqing, we found that only 0.55% were RhD-Negative. The information on the distribution characteristics of the RhD blood groups is important for a more reasonable blood collection program in Chongqing, especially during under-supplied.

According to previous reports, the incidence of many diseases was related to blood groups.^[13–15]^ The study by Ramesh which was conducted at various cancer hospitals in Kanpur and found that people with blood group A and AB had a lower risk of oral cancer than people with others.^[16]^ The Wolpin study found that people with blood group A, B, or AB had a higher risk of developing pancreatic cancer.^[17]^ Maybe, understanding the potential health risks of blood donors based on their blood type can help us expand our thinking on screening.

However, our study also has some limitations. First, there may be differences in ABO and RhD blood group distribution between the general population and blood donors. Second, we mainly assessed the Han nationality in Chongqing. Also, some people donate blood frequently that made the percentage is higher than other groups. So, we need to expand the scope of research to provide scientific-effective data on the distribution characteristics of ABO and RhD blood groups.

## 5 Conclusions

In conclusion, the distribution characteristics of the ABO and RhD blood group should be considered when improving blood collection program in Chongqing.

## Acknowledgments

We thank all of the blood donors and staff at the Chongqing Blood Center.

## Author contributions

**Data curation:** Hongmei Liao.

**Writing – original draft:** Hongmei Liao.

**Writing – review & editing:** Jun Li.
